# Supplementary material for: Human CD4 cytotoxic T lymphocytes mediate potent tumor control in humanized immune system mice
Source: Commun Biol. 2023 Apr 25;6:447. doi: 10.1038/s42003-023-04812-3 (PMC10130128; doi:10.1038/s42003-023-04812-3)
Supplement: Supplementary file 2 — Supplementary Information [file 42003_2023_4812_MOESM2_ESM.pdf]

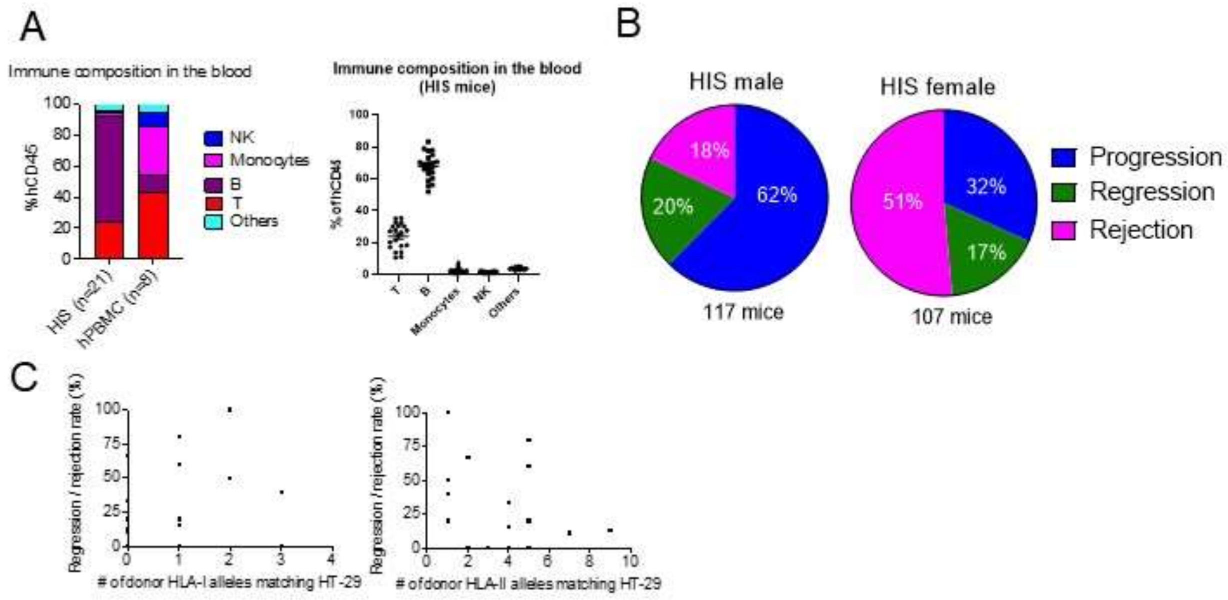

Supplementary figure 1 Characterization of the baseline immune composition and anti-tumor responses of the HT-29 HIS tumor model. (A) Immune composition in the blood of HIS mice 16 weeks after CD34$^+$ HSPC reconstitution. Human peripheral blood mononuclear cells (PBMCs) are shown for comparison. CD3 is used for identifying T cells, CD19 for B cells, CD14 for monocytes, and CD56 for NK cells. N=21 for HIS mice and n=8 for human PBMC. The results on the right are shown as means ± SEM. **(B)** Tumor growth outcome disaggregated by sex. $p < 0.0001$, Chi-square test. **(C)** The relationship between HSPC donor and HT-29 HLA compatibility and tumor growth outcome.

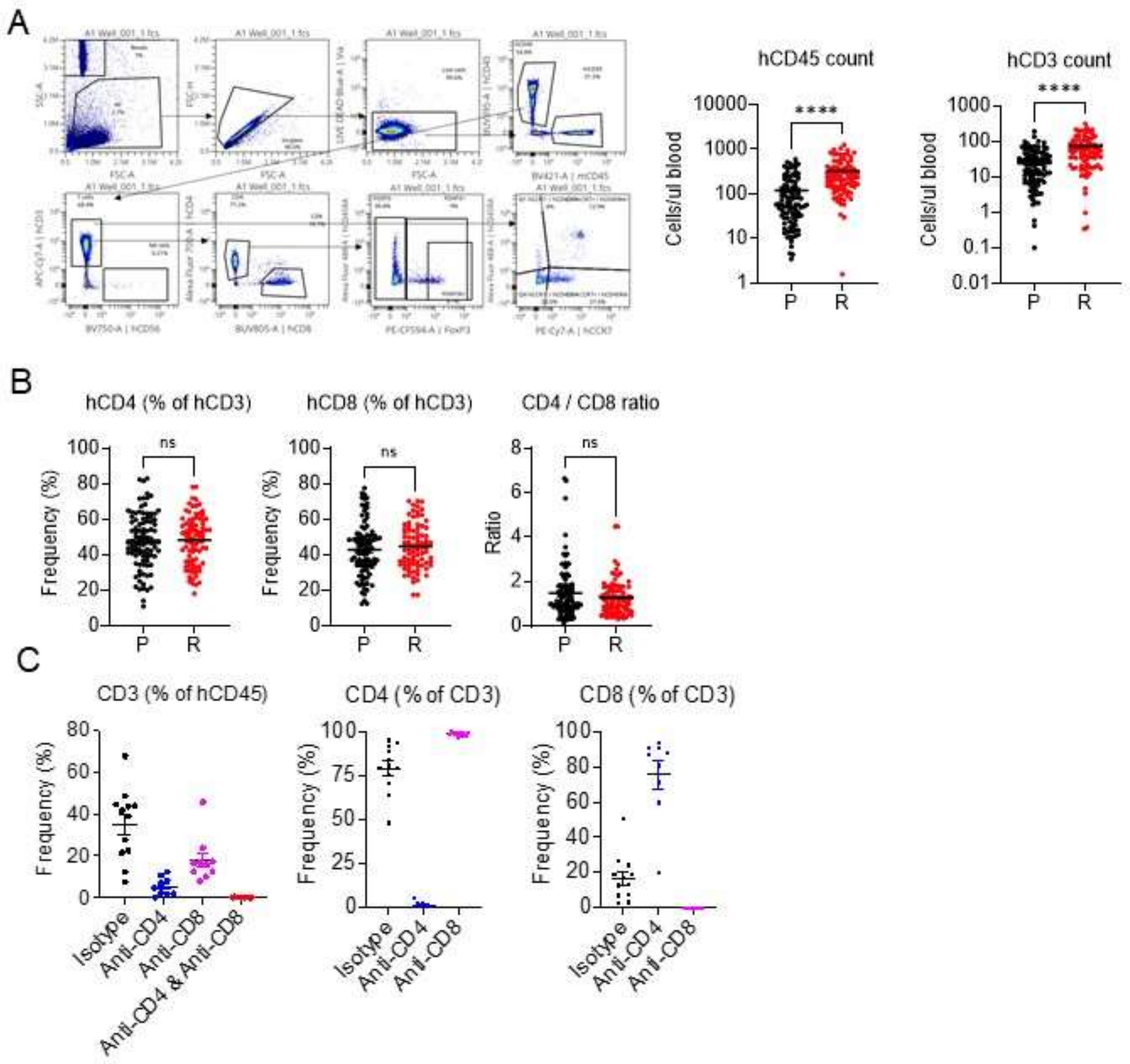

Supplementary figure 2 Pre-tumor immune cell phenotypes in the blood and T cell composition of the depletion experiment in figure 2. (A) Flow cytometry analysis gating strategy and correlation between pre-tumor hCD45 and hCD3 counts in the blood and tumor growth outcome. **(B)** Correlation between pre-tumor CD4 and CD8 frequencies and CD4 to CD8 ratio in the blood and tumor growth outcome. Correlation from P (n = 101) and R (n = 83) are shown. **(C)** Frequency of CD3, CD4, and CD8 T cells in the blood 28 days after tumor implantation. N=12 for the isotype, n=10 for the anti-CD8, n=9 for the anti-CD4, and n=9 for the anti-CD4 & anti-CD8 group. The results are shown as means ± SEM. ****$p < 0.0001$, unpaired t-test.

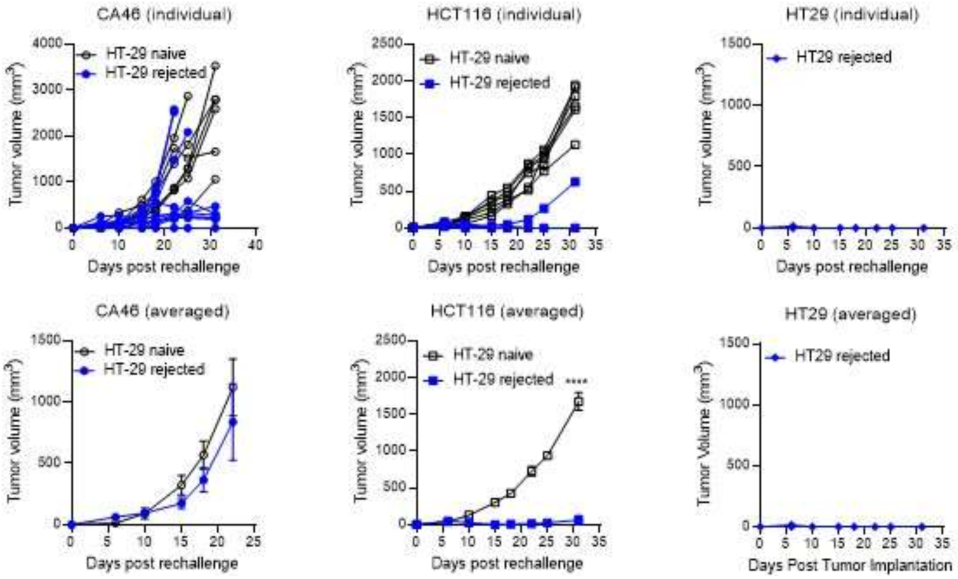

Supplementary figure 3 Tumor growth curves of mice that had previously rejected HT-29 and rechallenged with CA46, HCT116, or HT-29 tumor cell lines. Averaged tumor growth curves were plotted until the last time point when all mice were alive and shown as means ± SEM. N=7 for CA46 HT-29 naïve, n=10 for CA46 HT-29 rejected, n=6 for HCT116 HT-29 naïve, n=10 for HCT116 HT-29 rejected, and n=9 for HT-29 HT-29 rejected. Statistical significance was indicated for the last time point, ****$p < 0.0001$, two-way ANOVA.

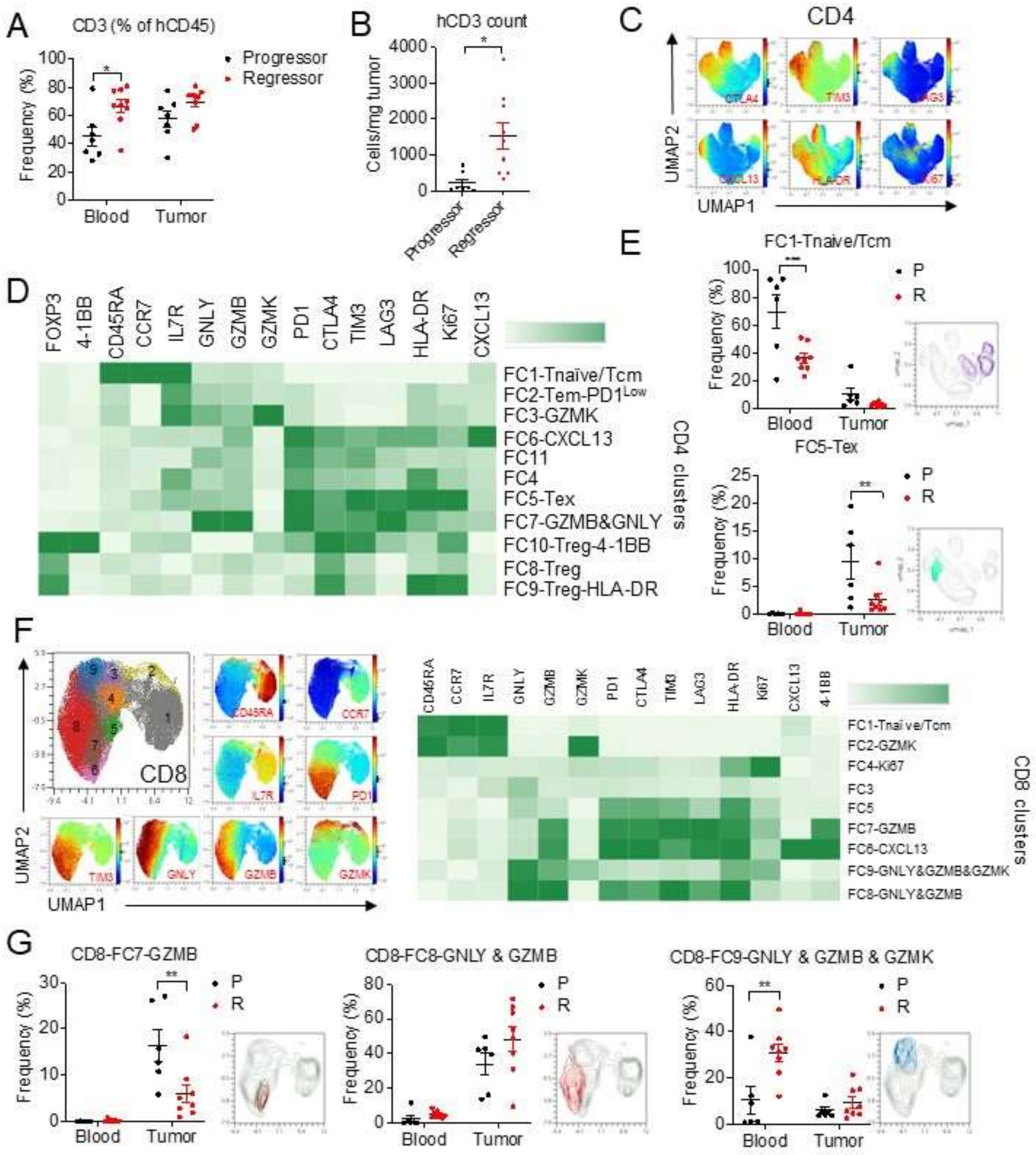

Supplementary figure 4 Human T cell phenotypes and correlations with tumor growth outcome by flow cytometry. (A-B) hCD3 T cell frequency and count in tumors and/or blood. N=6 for progressor and n=8 for regressor. **(C-D)** UMAP and heatmap of selected T cell lineage and functional markers across CD4 clusters. **(E)** Frequencies of the C1-Tnaïve/Tcm and C5-Tex (exhaustion) CD4 clusters in the blood and tumors of mice with progressing (n=6) and regressing (n=8) tumors. **(F)** CD8 clustering results as well as UMAP and heatmap of selected T cell lineage

and functional markers across clusters. **(G)** Frequencies of CD8 CTL clusters in the blood and tumors of tumor progressing (n=6) and regressing (n=8) mice. The results are shown as means ± SEM. *$p < 0.05$, **$p < 0.01$, ***$p < 0.001$, two-way ANOVA, Sidak's multiple comparison test for A, E, and G, unpaired t-test for B.

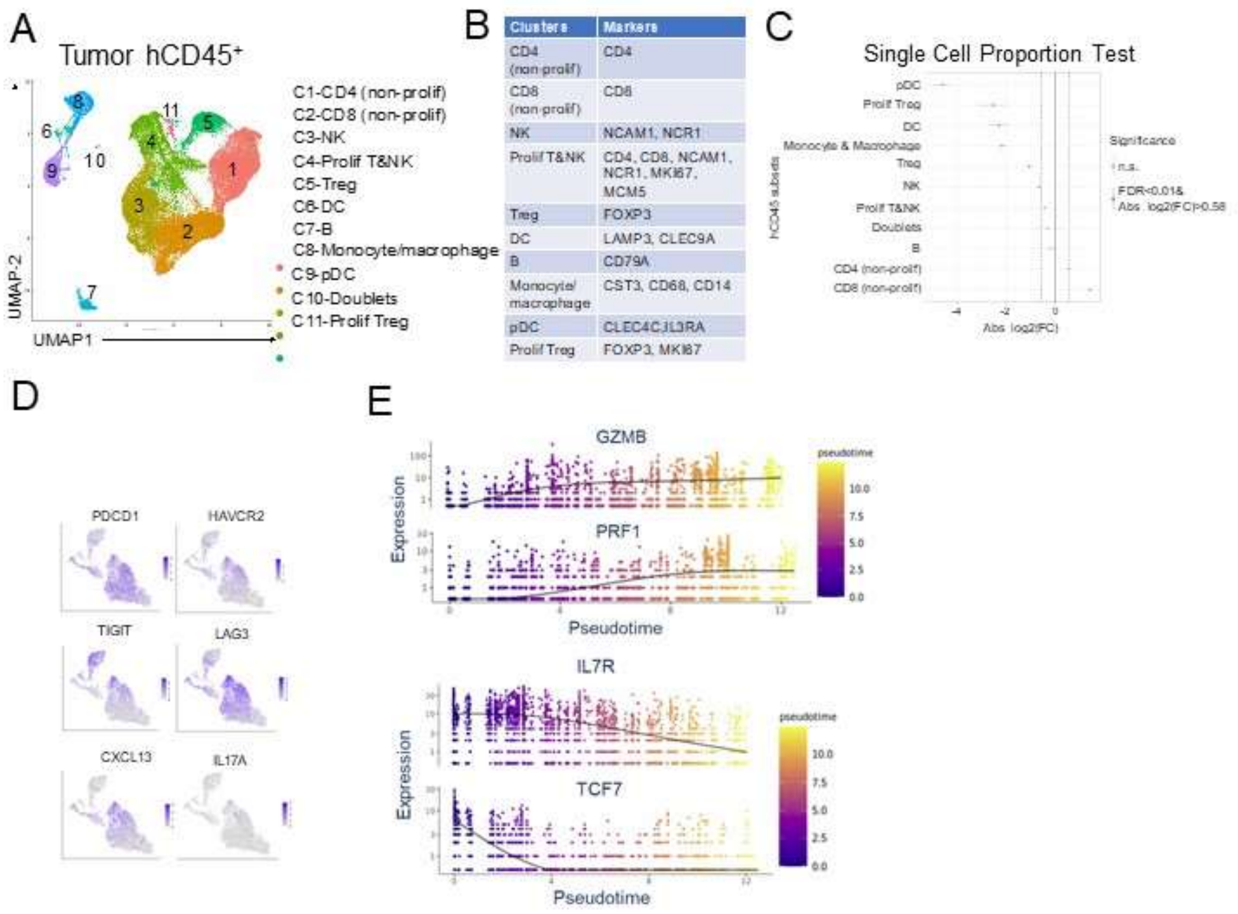

Supplementary figure 5 Human total immune cell and CD4 T cell phenotypes in the tumor by scRNAseq and TCRseq. (A) hCD45 clusters. **(B)** Differentially regulated markers used for hCD45 cluster annotation. **(C)** Comparison of the proportion of cells derived from progressing and regressing tumors in each hCD45 cluster. Analyses of (C) was performed using Single Cell Proportion Test. The dashed line indicates y = 0.58, which is equivalent to a fold change of 1.5, regressing over progressing tumors. A significant difference is defined by false discovery rate (FDR) < 0.01 and fold change > 1.5 or < -1.5. **(D)** UMAPs of selected T cell functional markers on CD4 T cell clusters. **(E)** Expression levels of markers for effector and naïve/central memory T cells over pseudotime.

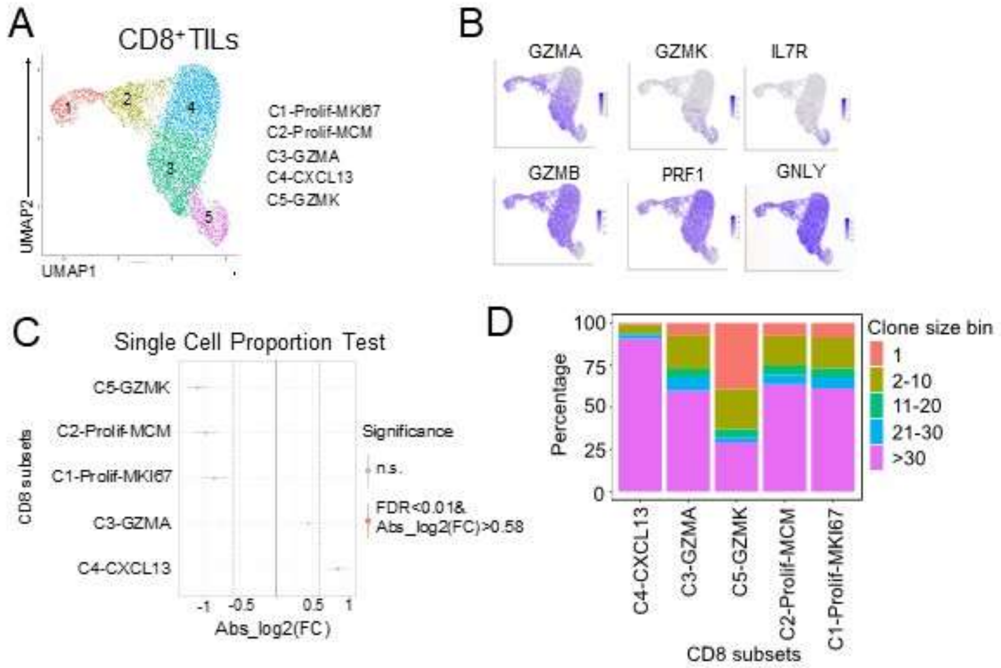

Supplementary figure 6 Human CD8 T cell phenotypes in the tumor and correlations with tumor growth outcome by scRNAseq and TCRseq. (A) CD8 clusters. **(B)** UMAP of selected T cell lineage and functional markers across CD8 clusters. **(C)** Comparison of the proportion of cells derived from progressing and regressing tumors in each CD8 cluster. **(D)** Percentage of TCRs of different clone size bins in each CD8 cluster. Analyses of C was performed using Single Cell Proportion Test. The dashed line indicates y = 0.58, which is equivalent to a fold change of 1.5, regressing over progressing tumors. A significant difference is defined by false discovery rate (FDR) < 0.01 and fold change > 1.5 or < -1.5.

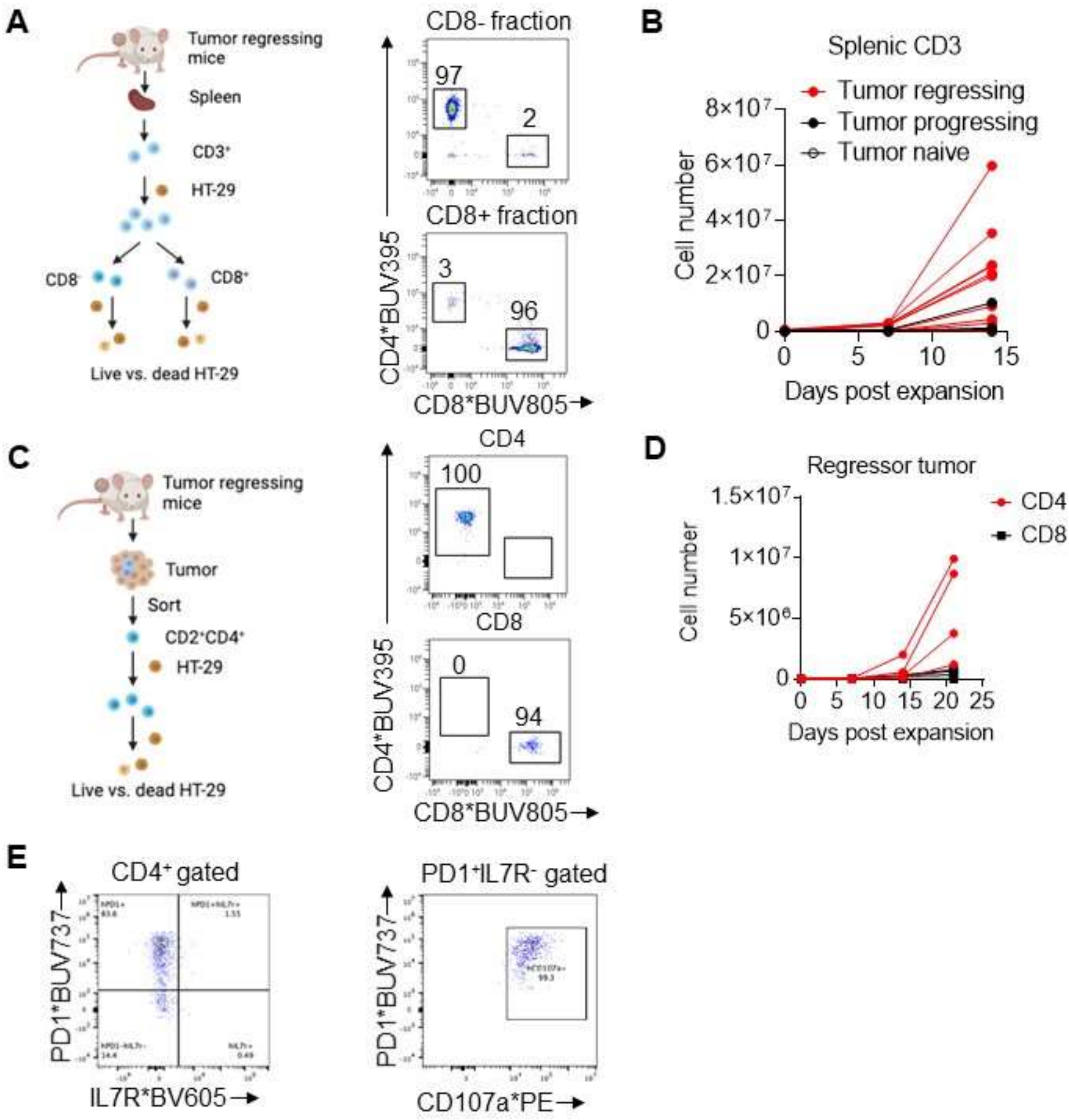

Supplementary figure 7 Schematic of killing assays, *ex vivo* expansion curves of T cells co-cultured with HT-29, and phenotypes of CD4 T cells from the killing assay (A) Schematic of killing assay using splenic T cells of tumor-regressing mice (created with BioRender.com). Purity after purification is shown. **(B)** *Ex vivo* expansion curve of splenic CD3^{+} T cells from tumor regressing (n = 10), progressing (n = 3) and naïve (n = 2) mice co-cultured with HT-29. **(C)** Schematic of killing assay using T cells from regressing tumors (created with BioRender.com). 75,000 CD2+CD4+ cells were obtained from the sort. Purity after expansion is shown. **(D)** *Ex vivo* expansion curve of regressing tumor-derived CD4^{+} and CD8^{+} T cells co-cultured with HT-29. N

= 4. **(E)** representative flow cytometry plots of PD1 and IL7R expression on CD4 T cells (left) and CD107a expression on $PD1^{+}IL7R^{-}$ CD4 T cells (right) at the end of the killing assay. The results of B and D are from repeated measurements of the same sample overtime.

Supplementary Table 1. Representative genes of CD4 clusters identified by scRNAseq

CD4 T cell cluster	Representative genes	Functional properties
C1-PRF1	CXCL13, CCL5, PRF1, CCL4, GNLY, GZMB, NKG7,	CTL
C2-IFIT3	ISG15, IFIT3, GZMB, GNLY, GZMA, GZMH	CTL
C3-KLRB1	KLRB1, IL17A, CTSH, CCR6	Th17
C4-IL7R	IL7R, GZMA	CTL
C5-Tnaive/Tcm	TCF7, CCR7, SELL, IL7R, LEF1, S1PR1, ANXA1	Tnaive/Tcm
C6-Prolif	MKI67, TYMS, MCM5, MCM7	Proliferating CD4 Tconv
C7-Treg	FOXP3, IL2RA, TNFRSF4, TIGIT, TNFRSF18, CTLA4	Treg
C8-Treg-TNFRSF9	FOXP3, IL2RA, TNFRSF4, TIGIT, TNFRSF18, CTLA4, TNFRSF9	Immune-suppressive Treg
C9-Treg-Prolif	FOXP3, IL2RA, TNFRSF4, TIGIT, TNFRSF18, CTLA4, TNFRSF9, MKI67, TYMS, MCM5, MCM7	Proliferating Treg

Supplementary Table 2. Frequencies of each CD4 T cell clusters of each mouse

	C1-PRF1 1580	C2-IFIT3 634	C3-KLRB1 308	C4-IL7R 1764	C5-Tnaive/Tcm 559	C6-Prolif 691	C7-Treg 618	C8-Treg-TNFRSF9 397	C9-Treg-Prolif 197
StRG47-Prog-hCD45-1	18.6	11.9	6.8	22.0	8.5	20.3	8.5	1.7	1.7
StRG47-Prog-hCD45-2	4.0	1.3	0.4	4.0	50.7	7.9	17.2	7.5	7.0
StRG47-Prog-hCD45-3	3.7	14.1	3.5	45.8	1.7	20.8	6.5	2.2	1.7
StRG47-Prog-hCD45-4	4.9	3.2	3.7	7.1	2.7	7.1	32.4	21.4	17.4
StRG47-Prog-hCD45-5	7.0	10.3	3.3	4.1	24.4	8.1	22.9	10.3	9.6
StRG47-Prog-hCD45-6	30.4	7.8	3.0	19.2	3.3	13.6	9.9	10.6	2.2
StRG47-Reg-hCD45-1	18.1	4.0	2.5	37.9	7.6	6.9	13.9	8.2	0.8
StRG47-Reg-hCD45-2	10.5	23.0	0.6	34.0	9.8	7.9	8.1	3.8	2.4
StRG47-Reg-hCD45-3	56.6	1.0	13.2	10.9	4.3	7.3	3.7	2.7	0.3
StRG47-Reg-hCD45-4	30.0	17.6	0.0	22.4	8.1	5.7	9.0	4.3	2.9
StRG47-Reg-hCD45-5	28.4	9.1	3.0	36.8	13.8	3.6	1.4	3.1	0.9

Supplementary Table 3. Flow cytometry antibody information

Target	Vender	Catalog number	Clone	Fluorochrome	Concentration (ng/ul)
EpCAM	BioLegend	324236	9C4	BV510	5
mCD45	eBioscience ™	58-0451-82	30-F11	AF532	2.5
hCD45	BD	563792	HI30	BUV395	2.5
CD2	BioLegend	300206	RPA-2.10	FITC	5
CD3	BioLegend	300318	HIT3a	APC-CY7	5
CD4	BioLegend	334328	A161A1	AF700	5
CD4	BioLegend	334328	5C3	AF700	5
CD8	BD	612889	SK1	BUV805	5
Foxp3	BD	563955	236A/E7	PE-CF594	5
CD45RA	BioLegend	304134	HI100	AF488	10
CCR7	BioLegend	353226	G043H7	PE-CY7	10
IL7R (CD127)	BD	748489 (OptiBuild)	HIL-7R-M21	BUV563	10
TIM3 (CD366)	BD	748820 (OptiBuild)	7D3	BUV615	10
CTLA4	BioLegend	369610	BNI3	BV605	5
PD1	BD	612791	EH12.1	BUV737	5
LAG3 (CD223)	BD	746609	T47-530	BV480	20
GranzymeB	BioLegend	515408	GB11	PacificBlue	5

GranzymeB	BioLegend	372212	QA16A02	PerCP-CY5.5	5
GNLY	eBioscience ™	12-8828-42	DH2	PE	10
GZMK	eBioscience ™	46-8897-42	G3H69	PerCP-eFluor 710	10
Ki67	BioLegend	350516	Ki-67	BV711	5
4-1BB	Biolegend	309808	4B4-1	PE-CY5	10
HLA-DR	Biolegend	307638	L243	BV570	5
CXCL13	R&D systems	IC801A	IC801A	APC	10
IL2	BD	612836	MQ1-17H12	BUV737	10
TNFa	BioLegend	502908	Mab11	PE	10
IFNg	BioLegend	502532	4S.B3	BV421	10
CD107a	BioLegend	328608	H4A3	PE	10
