# Supplementary material for: Human CD4 cytotoxic T lymphocytes mediate potent tumor control in humanized immune system mice
Source: Commun Biol. 2023 Apr 25;6:447. doi: 10.1038/s42003-023-04812-3 (PMC10130128; doi:10.1038/s42003-023-04812-3)
Supplement: Supplementary file 3 — Description of Additional Supplementary Files [file 42003_2023_4812_MOESM3_ESM.pdf]

Description of Additional Supplementary Files

File name: Supplementary Data 1

Description: HLA alleles of tumor cell lines used in this study.

File name: Supplementary Data 2

Description: Differentially upregulated gene sets of each CD4 cluster.

File name: Supplementary Data 3

Description: The source data behind the graphs in the paper.
