# Supplementary material for: Human CD4 cytotoxic T lymphocytes mediate potent tumor control in humanized immune system mice
Source: Commun Biol. 2023 Apr 25;6:447. doi: 10.1038/s42003-023-04812-3 (PMC10130128; doi:10.1038/s42003-023-04812-3)
Supplement: Supplementary file 7 — Reporting Summary [file 42003_2023_4812_MOESM7_ESM.pdf]

nature portfolio

Corresponding author(s): Yi-Fen Lu

Last updated by author(s): Apr 4, 2023

Reporting Summary

Nature Portfolio wishes to improve the reproducibility of the work that we publish. This form provides structure for consistency and transparency in reporting. For further information on Nature Portfolio policies, see our Editorial Policies and the Editorial Policy Checklist.

Statistics

For all statistical analyses, confirm that the following items are present in the figure legend, table legend, main text, or Methods section.

n/a	Confirmed	
☐	☒	The exact sample size (*n*) for each experimental group/condition, given as a discrete number and unit of measurement
☐	☒	A statement on whether measurements were taken from distinct samples or whether the same sample was measured repeatedly
☐	☒	The statistical test(s) used AND whether they are one- or two-sided<br>*Only common tests should be described solely by name; describe more complex techniques in the Methods section.*
☒	☐	A description of all covariates tested
☒	☐	A description of any assumptions or corrections, such as tests of normality and adjustment for multiple comparisons
☒	☐	A full description of the statistical parameters including central tendency (e.g. means) or other basic estimates (e.g. regression coefficient) AND variation (e.g. standard deviation) or associated estimates of uncertainty (e.g. confidence intervals)
☒	☐	For null hypothesis testing, the test statistic (e.g. *F*, *t*, *r*) with confidence intervals, effect sizes, degrees of freedom and *P* value noted<br>*Give P values as exact values whenever suitable.*
☒	☐	For Bayesian analysis, information on the choice of priors and Markov chain Monte Carlo settings
☒	☐	For hierarchical and complex designs, identification of the appropriate level for tests and full reporting of outcomes
☒	☐	Estimates of effect sizes (e.g. Cohen's *d*, Pearson's *r*), indicating how they were calculated

Our web collection on statistics for biologists contains articles on many of the points above.

Software and code

Policy information about availability of computer code

Data collection	Description of the custom code for data collection, including the version used, can be accessed through the following link: https://github.com/gchoonoo/WenLin_scs_2023
Data analysis	Description of the custom code for data analysis, including the version used, can be accessed through the following link: https://github.com/gchoonoo/WenLin_scs_2023

For manuscripts utilizing custom algorithms or software that are central to the research but not yet described in published literature, software must be made available to editors and reviewers. We strongly encourage code deposition in a community repository (e.g. GitHub). See the Nature Portfolio guidelines for submitting code & software for further information.

Data

Policy information about availability of data

All manuscripts must include a data availability statement. This statement should provide the following information, where applicable:

- Accession codes, unique identifiers, or web links for publicly available datasets
- A description of any restrictions on data availability
- For clinical datasets or third party data, please ensure that the statement adheres to our policy

SOURCE DATA is provided with this submission. The single-cell RNA sequencing and TCR sequencing data have been deposited in NCBI's Gene Expression Omnibus and are accessible through GEO Series accession number GSE223026 (https://www.ncbi.nlm.nih.gov/geo/query/acc.cgi?acc=GSE223026).

Human research participants

Policy information about studies involving human research participants and Sex and Gender in Research.

Reporting on sex and gender	N/A
Population characteristics	N/A
Recruitment	N/A
Ethics oversight	N/A

Note that full information on the approval of the study protocol must also be provided in the manuscript.

Field-specific reporting

Please select the one below that is the best fit for your research. If you are not sure, read the appropriate sections before making your selection.

☒ Life sciences ☐ Behavioural & social sciences ☐ Ecological, evolutionary & environmental sciences

For a reference copy of the document with all sections, see nature.com/documents/nr-reporting-summary-flat.pdf

Life sciences study design

All studies must disclose on these points even when the disclosure is negative.

Sample size	The sample size of most of our studies is determined by number of HIS mice generated per HSPC donor. The sample size per study has been shown sufficient by similar published tumor studies in mice.
Data exclusions	None
Replication	All in vitro studies were repeated 3 times. The tumor rejection phenotype was repeated in 223 HIS mice engrafted with human HSPC from 27 different donors.
Randomization	In the T cell depletion study, mice were randomized based on the frequencies and counts of human CD45+ and human CD3+ cells in the blood.
Blinding	Yes

Reporting for specific materials, systems and methods

We require information from authors about some types of materials, experimental systems and methods used in many studies. Here, indicate whether each material, system or method listed is relevant to your study. If you are not sure if a list item applies to your research, read the appropriate section before selecting a response.

Materials & experimental systems

n/a	Involved in the study	
☐	☒	Antibodies
☐	☒	Eukaryotic cell lines
☒	☐	Palaeontology and archaeology
☐	☒	Animals and other organisms
☒	☐	Clinical data
☒	☐	Dual use research of concern

Methods

n/a	Involved in the study	
☒	☐	ChIP-seq
☐	☒	Flow cytometry
☒	☐	MRI-based neuroimaging

Antibodies

Antibodies used	Relevant information, including supplier name, catalog number, and clone information, are provided in the MATERIALS AND MEOTHODS section.
Validation	All antibodies used for flow cytometry were validated in-house using human PBMC, mouse PBMC, and CD3/CD28 activated human T cells.

Eukaryotic cell lines

Policy information about cell lines and Sex and Gender in Research

Cell line source(s)	All cell lines were obtained from Regeneron tissue culture core.
Authentication	All cell lines were IMPACT tested negative for common human pathogens and profiled by RNA sequencing and HLA genotyping.
Mycoplasma contamination	All cell lines were tested negative for mycoplasma contamination.
Commonly misidentified lines (See ICLAC register)	None

Animals and other research organisms

Policy information about studies involving animals; ARRIVE guidelines recommended for reporting animal research, and Sex and Gender in Research

Laboratory animals	Relevant information, including species, strain, and age of the mice, are provided in the MATERIALS AND MEOTHODS section.
Wild animals	None
Reporting on sex	Spontaneous tumor regression/rejection was observed in both male and female mice and appeared more common in the female group. This result and gender info of a total of 223 mice can be found in supplementary figure 1C and SOURCE DATA.
Field-collected samples	None
Ethics oversight	All experiments involving mice were performed in compliance with all relevant ethical regulations and following protocols approved by the Regeneron Pharmaceuticals Institutional Animal Care and Use Committee (IACUC).

Note that full information on the approval of the study protocol must also be provided in the manuscript.

Flow Cytometry

Plots

Confirm that:

☒ The axis labels state the marker and fluorochrome used (e.g. CD4-FITC).

☒ The axis scales are clearly visible. Include numbers along axes only for bottom left plot of group (a 'group' is an analysis of identical markers).

☒ All plots are contour plots with outliers or pseudocolor plots.

☒ A numerical value for number of cells or percentage (with statistics) is provided.

Methodology

Sample preparation	See MATERIALS AND METHODS section.
Instrument	See MATERIALS AND METHODS section.
Software	See MATERIALS AND METHODS section.
Cell population abundance	The purity was determined by flow cytometry. Please see Supplementary figure 1A and 1C and their figure legend for details.
Gating strategy	For all flow cytometry experiments, debris, doubles, and dead cells were gated out using the FSC-A vs. SSC-A, FSC-A vs. FSC-H, viability dye vs. FSC-H gates, respectively. The boundaries between negative and positive populations were drawn based on biological negative control samples whenever possible. If such samples were not available, FMO negative controls were used.

☒ Tick this box to confirm that a figure exemplifying the gating strategy is provided in the Supplementary Information.
